# Advanced glycation end products reduce the calcium transient in cardiomyocytes by increasing production of reactive oxygen species and nitric oxide

**DOI:** 10.1002/2211-5463.12284

**Published:** 2017-09-26

**Authors:** Zeinab Hegab, Tamer M.A. Mohamed, Nicholas Stafford, Mamas Mamas, Elizabeth J. Cartwright, Delvac Oceandy

**Affiliations:** ^1^ Division of Cardiovascular Sciences The University of Manchester, Manchester Academic Health Science Centre UK; ^2^ J David Gladstone Research Institutes San Francisco CA USA; ^3^ Faculty of Pharmacy Zagazig University Egypt; ^4^ Keele Cardiovascular Research Group Institute of Science and Technology in Medicine Keele University Stoke‐on‐Trent UK

**Keywords:** advanced glycation end products, calcium, cardiomyocyte, nitric oxide, reactive oxygen species

## Abstract

Advanced glycation end products (AGE) are central to the development of cardiovascular complications associated with diabetes mellitus. AGE may alter cellular function through cross‐linking of cellular proteins or by activating the AGE receptor (RAGE). However, the signalling molecules involved during AGE stimulation in cardiomyocytes remain unclear. Here, we investigated the effects of AGE treatment on intracellular calcium homeostasis of isolated cardiomyocytes and studied the activation of signalling molecules involved in this process. Treatment of cardiomyocytes with AGE for 24 h resulted in a dose‐dependent reduction in calcium transient amplitude, reaching a maximum 50% reduction at a dose of 1 mg·mL^−1^. This was accompanied with a 32% reduction in sarcoplasmic reticulum calcium content but without any detectable changes in the expression of major calcium channels. Mechanistically, we observed a significant increase in the production of reactive oxygen species (ROS) in AGE‐treated cardiomyocytes and enhancement of NADPH oxidase activity. This was accompanied with activation of p38 kinase and nuclear translocation of NF‐κB, and subsequently induction of inducible NO synthase (iNOS) expression, leading to excessive nitric oxide production. Overall, our data reveal the molecular signalling that may underlie the alteration of intracellular calcium homeostasis in cardiac myocytes due to AGE stimulation. This may provide new insights into the pathophysiological mechanisms of the development of diabetic cardiomyopathy.

**Abbreviations**
AGE: advanced glycation end product
DPI: diphenyleneiodonium
iNOS: inducible NO synthase
LTCC: L‐type calcium channel
MAPK: mitogen‐activated protein kinase
NADPH: nicotinamide adenine dinucleotide phosphate
NCX: sodium calcium exchanger
NF‐κB: nuclear factor kappa B
NO: nitric oxide
NRCM: neonatal rat cardiomyocytes
PDTC: pyrrolidine dithiocarbamate
PLB: phospholamban
PMCA: plasma membrane calcium ATPase
RAGE: receptor of AGE
ROS: reactive oxygen species
RyR: ryanodine receptor
SR: sarcoplasmic reticulum

The prevalence of diabetes is increasing with the projected prevalence of diabetes worldwide and is escalating from 285 million in 2010 to 439 million in 2030 1. Cardiovascular complications are the principle causes of morbidity and mortality in patients with diabetes, accounting for up to 68% of diabetic fatalities 2. These include atherosclerosis, coronary heart disease, congestive heart failure and diabetic cardiomyopathy. The latter refers to a condition where diabetic condition alters cardiac structure and function directly without affecting the vasculature and independent of the occurrence of hypertension or valvular disease 3.

Diabetic cardiomyopathy has been associated with both type 1 and type 2 diabetes and is characterized by early‐onset diastolic dysfunction followed by systolic dysfunction 4. It is believed that both structural and functional changes play significant roles in the process. For example, the extent of cardiac fibrosis is much greater in both animal models and human patients with diabetic cardiomyopathy 5. Furthermore, diabetes may also impair cardiomyocyte excitation–contraction coupling and hence alter intracellular calcium homeostasis 6, 7, possibly explaining the reduction in cardiac contractile function in diabetic cardiomyopathy.

However, the mechanism of how prolonged hyperglycaemia can cause cardiomyopathy is not completely understood. One of the possible factors that may be involved is the production and accumulation of advanced glycation end products (AGEs) 8. AGEs are heterogenous group of molecules resulting from the nonenzymatic glycation and oxidation of proteins and lipids in the presence of reducing sugars 9. Accumulating evidence showed crucial contribution of AGEs in the evolution and progression of heart failure in patients with diabetes as clinical studies have found correlation between serum AGE levels with severity of heart failure and coronary heart disease 10, 11 as well as with systolic and diastolic cardiac dysfunction 12, 13.

It is understood that the cardiac effects of AGEs may be through cross‐linking of extracellular matrix and/or activation of the AGE receptor (RAGE) (reviewed in 14). AGEs may cross‐link with extracellular matrix proteins such as collagen, laminin, and elastin leading to the impaired degradation and increased stiffness 15. On the other hand, AGE may affect intracellular signalling pathways via activation of RAGE. RAGE signalling has been widely investigated in vascular endothelial and smooth muscle cells as well as in macrophages 16; however, its role in the cardiomyocytes is not completely understood. Here, we investigated the direct effects of AGE stimulation on cardiomyocytes focusing on examining the effects of AGE stimulation on the intracellular calcium dynamics as well as investigating the signalling molecules that may be affected by AGE stimulation.

## Materials and methods

### Isolation and culture of rat primary neonatal cardiomyocytes

Neonatal rat cardiomyocytes were isolated from two‐ to three‐day‐old of Sprague Dawley rat neonates using the protocol described previously 17, 18. All experiments involving animals were performed in accordance with the United Kingdom Animals (Scientific Procedures) Act 1986 and were approved by the University of Manchester Ethics Committee. For NRCMs isolation, extracted neonatal hearts were preserved into ice‐cold filter‐sterilized ADS solution (116 mm NaCl, 20 mm HEPES, 1 mm NaH_2_PO_4_, 5.5 mm glucose, 5.5 mm KCl, 1 mm MgSO_4_, pH 7.35 adjusted with NaOH). The top section of the heart was excised to remove the atria. Then, the remaining part of the hearts were cut into half and were incubated in ADS solution containing 0.6 mg·mL^−1^ collagenase A (Roche Applied Bioscience, Mannheim, Germany) and 0.6 mg·mL^−1^ pancreatin (Sigma‐Aldrich, St. Louis, MO, USA) at 37 °C for 7 min in a shaking water bath. After seven successive cycles of digestion, cells pooled from all digestions were centrifuged at 335 ***g*** for 5 min. Cells were plated onto 100‐mm Falcon tissue culture dishes for 90 min in preplating medium (68% DMEM, 17% M199 medium, 10% horse serum, 5% FBS and 2.5 μg·mL^−1^ amphotericin B) to attach cardiac fibroblasts. Most of the cardiomyocytes remained floating and were collected afterwards. Cardiomyocytes were then plated on BD Falcon Primaria plates or on laminin‐coated cover slips in plating medium containing 68% DMEM, 17% M199 medium, 10% horse serum, 5% FBS, 2.5 μg·mL^−1^ amphotericin B and 1 μm BrdU (5‐bromo‐2‐deoxyuridine). The following day, NRCMs were washed twice using PBS and then cultured in maintenance media (80% DMEM, 20% M199, 1% FBS, 2.5 μg·mL^−1^ amphotericin B and 1 μm BrdU) at 37 °C.

### Intracellular calcium analysis

The fluorescent calcium indicator Indo‐1 AM (Molecular Probes, Eugene, OR, USA) was used to measure cytosolic free calcium. NRCMs were plated on laminin‐coated glass coverslips. Cells were loaded with 5 μm Indo‐1 AM for 15 min at 37^o^ C in the dark. The cells were then washed with maintenance media and incubated for further 30 min at 37 °C to de‐esterify the dye completely. The coverslip was placed in a bath on the stage of an epifluorescence‐adapted inverted Olympus IX70 microscope connected to an Olympus America camera. The myocytes were continuously perfused with Tyrode solution (131 mm NaCl, 4 mm KCl, 1 mm CaCl_2_, 1 mm MgCl_2_, 10 mm glucose, 10 mm HEPES, pH 7.4 adjusted with NaOH). The MyoPacer field stimulator (Ionoptix Inc., Westwood, MA, USA) was used for field stimulation of the cardiomyocytes through two silver wire electrodes initiating an electrical current at a frequency of 1 Hertz (Hz). Calcium changes occurring during cardiomyocyte contraction were recorded at 37 °C under basal conditions or after stimulation with glycated bovine serum albumin (AGE‐BSA, Calbiochem, EMD Millipore, Billerica, MA, USA) at 0.1–1 mg·mL^−1^ for 24 h. For SR calcium content analysis, calcium transient was measured after superfusion with 10 mm caffeine in the absence of electrical field stimulation. The amplitude of the resulting transient was used as a measure for the SR calcium content. For analysing the involvement of NF‐κB, cells were treated with 100 nm of NF‐κB inhibitor, pyrrolidine dithiocarbamate (PDTC), for 1 h prior to stimulation with AGE. To examine the role of NADPH oxidase, we treated NRCM with 1 μm NADPH oxidase inhibitor, diphenyleneiodonium (DPI), for 1 h prior to stimulation with AGE.

### Western blot

Western blots to analyse protein expression were conducted using the standard protocol as described previously 17, 18. Primary antibodies used were anti‐L‐type Ca^2+^ channel (Abcam, Cambridge, UK), anti‐ryanodine receptor (Abcam), anti‐SERCA2a (Santa Cruz Biotechnology, Dallas, TX, USA), anti‐NCX (Cell Signaling, Danvers, MA, USA), anti‐phospho‐PLB (Millipore, Bellerica, MA, USA), anti‐total‐PLB (Millipore), anti‐PMCA1 (Abcam), anti‐PMCA4 (Abcam), anti‐RAGE (Abcam), anti‐phospho‐p38 (Cell Signaling), anti‐total‐p38 (Cell Signaling), anti‐iNOS (Abcam), anti‐α‐tubulin (Calbiochem) and anti‐GAPDH (Santa Cruz). For visualization, we used HRP‐conjugated secondary antibodies, which were obtained from either Cell Signaling or Dako.

### Cell viability and caspase 3/7 analyses

To examine whether AGE treatment induces cell death and/or apoptosis, we treated NRCMs with 1 mg·mL^−1^ AGE for 24 h and performed MTT and caspase 3/7 assays. For the MTT assay, NRCMs were plated in 24‐well plates at a density of 3 × 10^5^ cells per well. Following 24‐h treatment with AGE, 0.45 mg·mL^−1^ thiazolyl blue tetrazolium bromide (Sigma) was added to each well and cells were incubated for 1 h at 37 °C. Formazan crystals were then dissolved by adding 100 μL of a solubilization solution containing 0.1N HCl in isopropanol, and cell viability was analysed by measuring the absorbance at 570 nm on a spectrophotometer.

For analysing caspase 3/7 activity, cells were plated at a density of 3 × 10^5^ and treated with AGE for 24 h. Following treatment, cells were lysed for 30 min through the addition of 100 μL of cell culture lysis reagent (Promega, Madison, WI, USA). To assess caspase activity, 20 μL of lysate was mixed with an equal volume of caspase‐Glo 3/7 reagent (Promega) and incubated for 1 h at room temperature. Luminescence was measured using a Lumat luminometer (Berthold Technologies, Bad Wildbad, Germany).

### Immunofluorescence

Immunofluorescence analyses were conducted using the protocol as described elsewhere 17, 18. To detect NFκB subcellular localization, we used anti‐NFκB (p65) (Santa Cruz) and anti‐α‐actinin (Sigma) as primary antibodies and detected using fluorescent‐labelled secondary antibodies (Jackson Lab, West Grove, PA, USA). DAPI was used to stain the nuclei. To examine S‐nitrosylation of RyR and SERCA2a, we used anti‐SERCA2a (Santa Cruz Biotechnology), anti‐RYR (Abcam) and anti‐S‐nitrosocysteine (Abcam).

### NF‐κB luciferase assay

We used an adenovirus‐containing luciferase reporter driven by NFκB elements. The generation of the virus has been described in previous publication 17. NRCMs were transduced with the virus 24 h prior to treatment with the AGE. Luciferase activity was detected 24 h following AGE treatment using luciferase detection reagent (Promega).

### Detection of intracellular reactive oxygen species

Intracellular reactive oxygen species (ROS) level was assessed using 2,7‐dichlorodihydrofluorescein diacetate (DCF‐DA) dye (Molecular Probes). For this procedure, NRCMs were plated on laminin‐coated glass bottom black‐walled 24‐well plates at a concentration of 10^6^ cells per well. Following stimulation with AGE, cells were incubated with DCF‐DA (10 μm) in PBS at 37 °C for 30 min in the dark. PBS was discarded and replaced by maintenance media and the cells were immediately used for ROS measurement. Fluorescence was detected using a live cell imaging Leica AS MDW inverted fluorescent microscope where the cells were kept at 37 °C.

### NADPH oxidase assay

For this assay, NRCMs were harvested in PBS. After single washing step, the cells were resuspended in 1 mm EGTA, 20 mm KH_2_PO_4_, 25 mg·mL^−1^ leupeptin, 10 mg·mL^−1^ aprotinin, and 1 mm phenylmethanesulfonyl fluoride. NRCMs were lysed in this medium by multiple pipetting up and down on ice. A luminescence assay was used to monitor NADPH oxidase activity in the cell homogenates. 100 μL of tissue homogenates was added to 900 μL of 50 mm phosphate buffer, pH 7.0 (1 mm EGTA, 150 mm sucrose, 500 mm lucigenin, and 100 mm NADPH), where lucigenin is an electron acceptor and NADPH is the substrate. NADPH oxidation into NADP by NADPH oxidase requires an electron transfer. Lucigenin is an electron acceptor and therefore becomes reduced by the release of electron. This reaction emits light. Photoemission was measured every minute for 14 min using a luminometer (Berthold Technologies). The amount of emitted light is used as an indicator of NADPH oxidase activity. The assay was repeated on cell homogenates from AGE‐treated cells but with the addition of 25 μm diphenyleneiodonium (DPI) (NADPH oxidase inhibitor) per reaction mixture. All used reagents were purchased from Sigma‐Aldrich.

### Detection of intracellular nitric oxide bioavailability

We used nitric oxide (NO)‐sensitive fluorescence dye DAF‐FM (4‐amino‐5‐methylamino‐2′,7′‐difluorofluorescein) (Molecular Probes) to measure NO bioavailability. Cultured NRCMs following treatment with AGE were loaded with 10 μm DAF‐FM for 30 min at 37 °C. The medium containing the dye was discarded and then replaced with maintenance medium containing 1 mm L‐arginine (Sigma‐Aldrich) and incubated for further 45 min at 37 °C for complete de‐esterification of the dye. Fluorescent signal was detected using a live cell imaging Leica AS MDW inverted fluorescent microscope where the cells were kept at 37 °C.

### Data analysis

Data are expressed as mean ± SEM. Student's *t*‐test or one‐way ANOVA followed by post hoc multiple comparison was used where appropriate. The probability level for statistical significance was set at *P* < 0.05.

## Results

### AGE reduces intracellular calcium transient in cardiomyocytes

**Figure 1:**
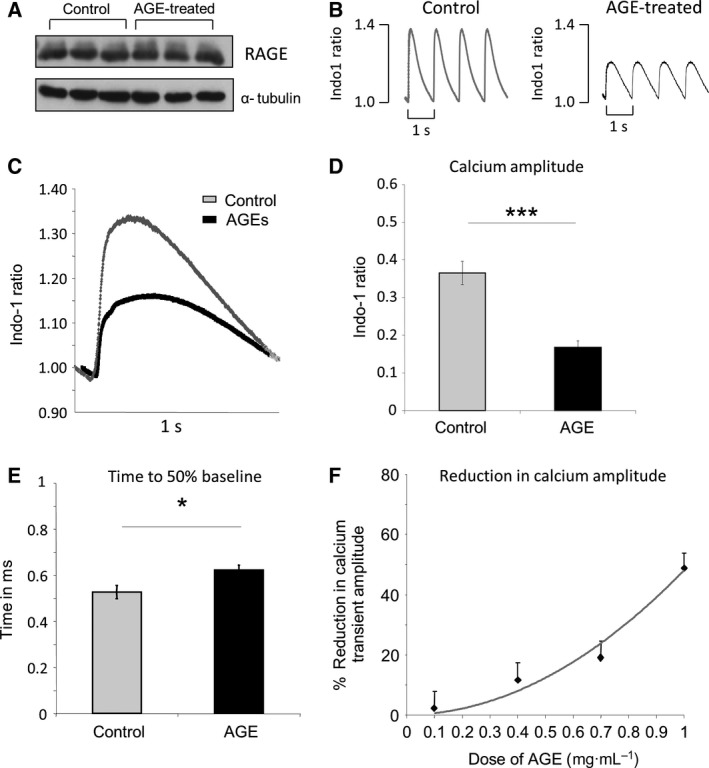
Advanced glycated end products (AGEs) reduce intracellular calcium transient in isolated cardiomyocytes. (A) Representative western blots showing the expression of AGE receptor (RAGE) and loading control (α‐tubulin) in neonatal rat cardiomyocytes (NRCM) with or without treatment with AGE (1 mg/mL for 24 h). (B) Representative intracellular calcium traces obtained from NRCM showing reduction in calcium transient amplitude and decay rate following AGE treatment (1 mg/mL for 24 h). Cells were loaded with Indo‐1 and field‐stimulated at a frequency of 1 Hz. (C) Average trace of calcium transients from control and AGE‐treated cardiomyocytes (1 mg/mL for 24 h). (D) Quantification of calcium amplitude and (E) time to 50% baseline showed a significant decrease in calcium amplitude (*P* < 0.001) and significant prolongation of time to 50% baseline (*P* < 0.01) (*n* = 18 cells from three independent NRCM preparations). (F) Dose‐dependent reduction in calcium transient amplitude in NRCMs treated with 0.1–1 mg/mL AGE for 24 h (*n* = 8–18 cells).

We used primary cardiomyocytes isolated from rat neonates (NRCM) to study the effects of AGE treatment on intracellular calcium transient. NRCM expressed AGE receptor (RAGE) as shown by western blot analysis (Fig. 1A), suggesting that this cell is a good model to analyse the effect of AGE. Cultured NRCMs were treated with glycated bovine serum albumin (AGE‐BSA) at a concentration of 1 mg·mL^−1^ for 24 h. No change in RAGE expression was observed following AGE treatment (Fig. 1A). Intracellular calcium was measured using Ca^2+^ probe Indo‐1 and field‐stimulated at 1 Hz. AGE treatment significantly reduced Ca^2+^ amplitude by ~50% (Fig. 1B–D). The rate of Ca^2+^ decay was also significantly prolonged as indicated by analysis of time to 50% baseline (Fig. 1E). Furthermore, when we treated NRCM with various doses of AGE ranging from 0.1 to 1 mg·mL^−1^, we found that the effect of AGE treatment in reducing Ca^2+^ amplitude was dose dependent (Fig. 1F).

### AGE reduces SR calcium content

**Figure 2:**
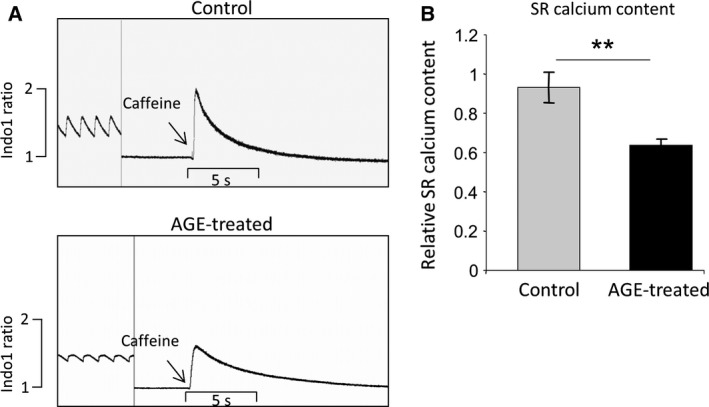
Advanced glycation end products reduce sarcoplasmic reticulum (SR) calcium content. (A) Example of calcium traces showing the response to 10 mm caffeine in control and AGE‐treated NRCM (1 mg/mL, 24 h). Field stimulation was stopped prior to caffeine induction. (B) Quantification of fluorescent signal suggested a significant decrease in SR calcium content (*P* < 0.01) in AGE‐treated cells compared to control (*n* = 9–11 cells from three independent preparations).

One major determinant of the reduction in Ca^2+^ transient amplitude is the level of Ca^2+^ in the intracellular Ca^2+^ storage, that is the sarcoplasmic reticulum (SR) Ca^2+^ content. To determine the SR Ca^2+^ content, we treated AGE‐stimulated and control cells with 10 mm caffeine in the absence of field stimulation. Data shown in Fig. 2A,B indicated that the SR Ca^2+^ content was significantly reduced in AGE‐treated cells by ~32%, which may contribute to the decline in the Ca^2+^ transient amplitude described above.

### Expression of calcium‐handling molecules are not altered by AGE treatment

**Figure 3:**
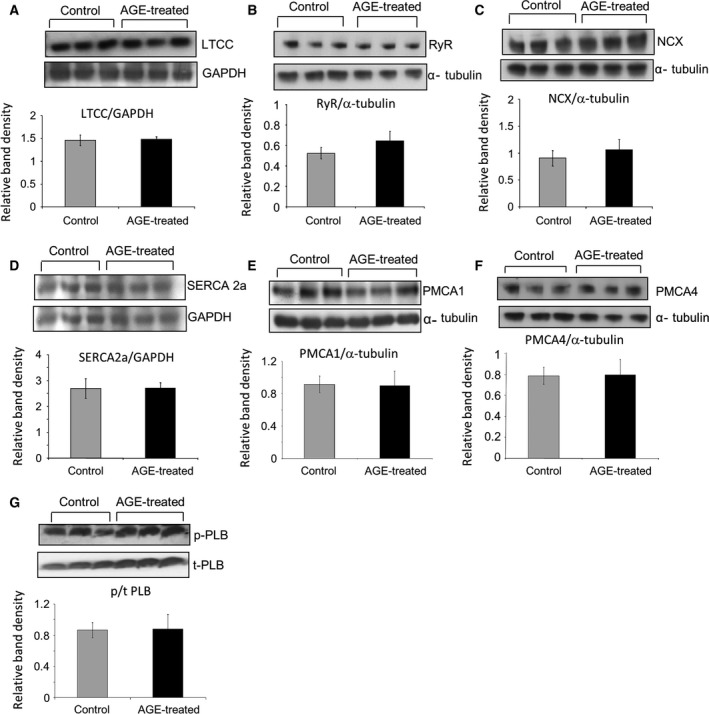
Advanced glycation end product treatment does not alter expression of major calcium channels. Representative western blots of (A) L‐type calcium channel (LTCC), (B) ryanodine receptor (RyR), (C) sodium/calcium exchanger (NCX), (D) sarcoplasmic reticulum ATPase 2a (SERCA2a), (E) plasma membrane calcium ATPase 1 (PMCA1), (F) PMCA4 and (G) phosphorylated and total phospholamban (p/t‐PLB) accompanied with loading controls (GAPDH/α‐tubulin). Quantification of band densities as described by the bar graphs suggested that there was no significant change in protein expressions following AGE treatment (1 mg/mL, 24 h; *n* = 6–8).

Next, we examined whether the change in intracellular Ca^2+^ dynamic in AGE‐treated NRCM was due to changes in the expression levels of Ca^2+^ handling proteins. Protein extracts from NRCM treated with 1 mg·mL^−1^ AGE for 24 h and control cells were subjected to western blot analysis to measure expression of ryanodine receptor (RyR), SERCA2a, L‐type Ca^2+^ channel (LTCC), sodium calcium exchanger (NCX), isoform 1 and 4 of the plasma membrane calcium ATPase (PMCA1 and PMCA4) as well as phosphorylated and total phospholamban (PLB). Western blots and subsequent quantification of band density showed that there was no difference in the expression levels of Ca^2+^ handling proteins following treatment with AGE (Fig. 3A–G). These data suggested that the effect of AGE treatment was mainly due to alteration of protein function rather than level of expression.

### Induction of reactive oxygen species formation by AGE

**Figure 4:**
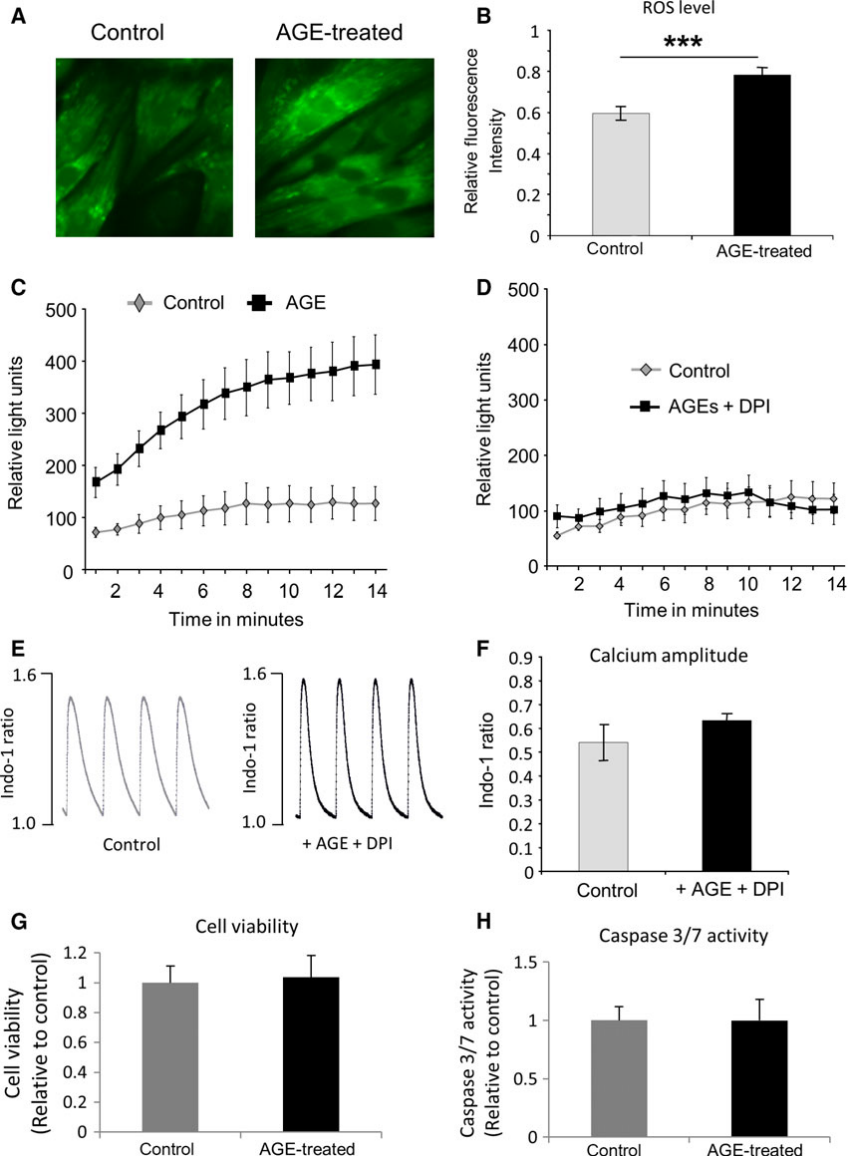
Advanced glycation end products induce intracellular reactive oxygen species (ROS) level and NADPH oxidase activity. (A) Images of control and AGE‐treated NRCMS stained with ROS‐sensitive dye DCF‐DA. (B) Quantification of fluorescence intensity indicated a significantly higher ROS level in NRCM treated with AGE (1 mg/mL, 24 h) (*P* < 0.001, *n* = 38–48 cells). (C) NADPH oxidase activity measurement in AGE‐treated and control NRCMs showed that AGE‐treated NRCM exhibited a significant increase (*P* < 0.01) in NADPH oxidase activity compared to the control cells (*n* = 7–9). (D) Addition of NADPH oxidase inhibitor diphenyleneiodonium (DPI, 25 μm) inhibited the difference between control and AGE‐treated NRCM (*n* = 5). (E) Examples of calcium traces from control and AGE‐treated NRCMs (1 mg/mL, 24 h) in the presence of 1 μm DPI. (F) Quantification of calcium amplitude showed no difference between control and AGE + DPI‐treated NRCM, indicating that DPI treatment suppressed the effect of AGE. (G) NRCM viability following treatment with AGE (1 mg/mL, 24 h) was assessed using MTT assay. There was no difference in the MTT colorimetric signal intensity between control and AGE‐treated cells (*n* = 12 in each group), suggesting that there was no difference in cell viability between these groups. (H) Consistently, analysis of caspase 3/7 activity showed that there is no difference in apoptosis level between AGE‐treated and control cells (*n* = 12 in each group).

It has been reported that in endothelial and hepatic stellate cells, RAGE activation by AGE may induce the formation of reactive oxygen species (ROS) via activation of NADPH oxidase 19, 20. To investigate whether ROS level was elevated in NRCM following AGE exposure, we used a ROS‐sensitive dye (DCF‐DA) to measure intracellular ROS. We found a significant increase in intracellular ROS level in AGE‐treated NRCM as indicated in Fig. 4A,B.

We then examined whether NADPH oxidase activity was increased in these cells. NADPH oxidase assay was performed on fresh cell lysates of AGE‐treated and control NRCM. A significant increase in NADPH oxidase activity was detected in AGE‐treated NRCM compared to control cells (Fig. 4C). Furthermore, addition of NADPH oxidase inhibitor diphenyleneiodonium (DPI, 25 μm) inhibited the difference between AGE‐treated and control NRCM (Fig. 4D), strongly supporting the idea that AGE treatment increased NADPH oxidase activity.

### NADPH oxidase may be involved in AGE‐induced intracellular calcium changes

To further analyse whether elevated NADPH oxidase was responsible for the changes in intracellular Ca^2+^ dynamics, we treated NRCM with 1 μm DPI for 1 h before adding AGE (1 mg·mL^−1^) for a further 24 h. We then analysed the Ca^2+^ transient in comparison with nontreated NRCM. As described in Fig. 4E,F, there was no difference in Ca^2+^ transient amplitude between these groups of NRCM, suggesting that DPI treatment might suppress the effect of AGE on NRCM. Taken together, the data above indicated that NADPH oxidase was involved in mediating the effects of AGE treatment on cardiomyocytes.

### NRCM viability following AGE treatment

As the ROS level was significantly increased in AGE‐treated NRCM, it was important to examine whether this affected cell viability and/or apoptosis. Therefore, we performed an MTT assay and analysed caspase 3/7 activity in NRCM treated with 1 mg·mL^−1^ AGE for 24 h. We found that there was no significant difference in cell viability or apoptosis levels between AGE‐treated and control cells as indicated by MTT and caspase 3/7 assay, respectively (Fig. 4G,H).

### p38 kinase and NFκB are activated by AGE

**Figure 5:**
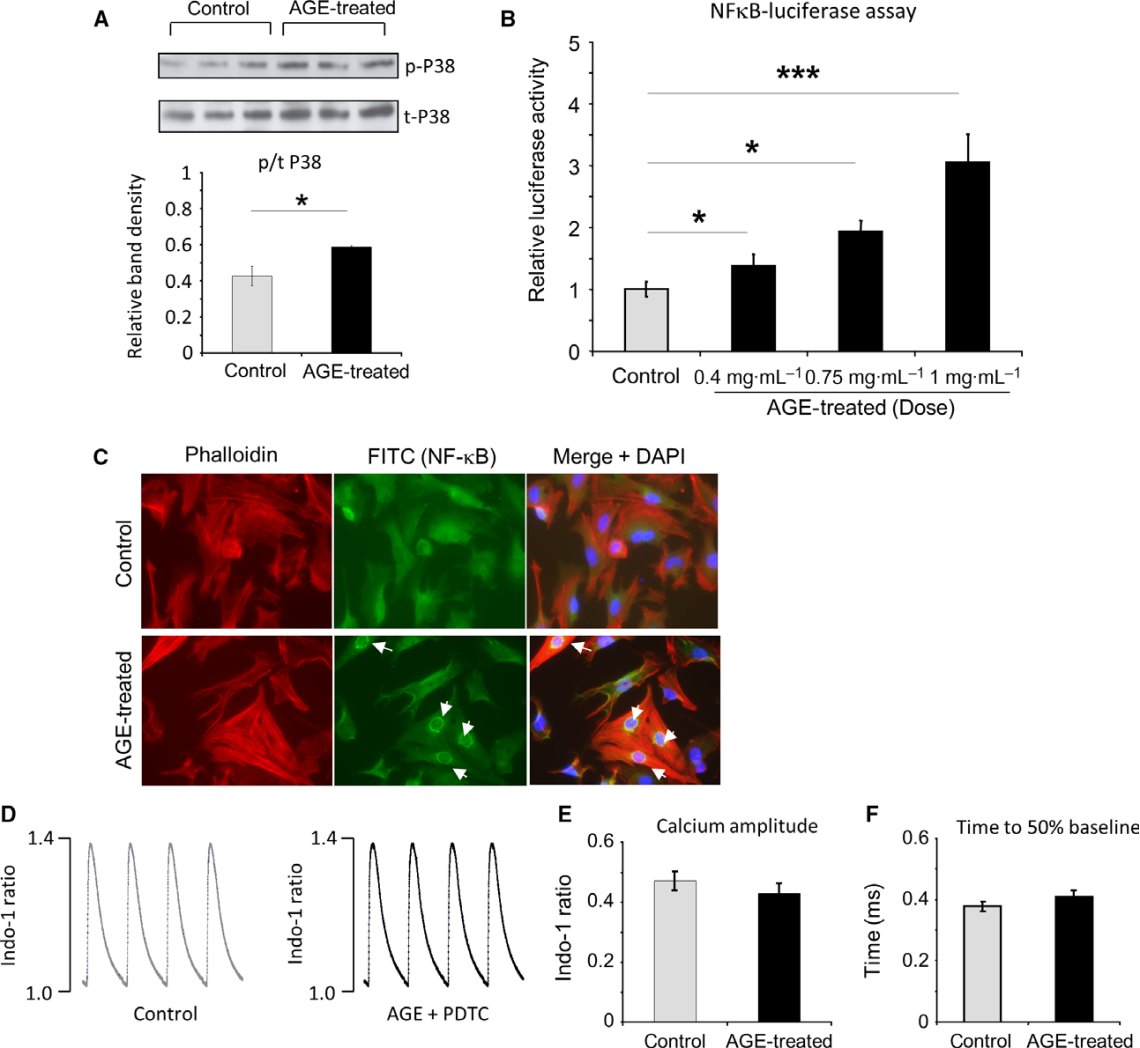
Treatment with AGE induces p38 MAPK and NFκB activation in NRCM. (A) Western blots of phosphorylated and total p38 kinase and quantification of band density showed a significant increase in phosphorylated/total p38 level in AGE‐treated NRCM (*P* < 0.05, *n* = 6). (B) Assessment of NFκB activity using NFκB luciferase reporter construct indicated a dose‐dependent increase in NFκB activity following AGE treatment (0.4–1 mg/mL, 24 h, *P < 0.05, ***P < 0.001, *n* = 9 in each groups). (C) Immunofluorescent staining of AGE‐treated (1 mg/mL, 24 h) and control NRCM. Cells were stained with anti‐NFκB antibody (green), phalloidin (red) and DAPI (blue). AGE treatment induced NFκB nuclear translocation. (D) Representative calcium traces of control and AGE‐treated NRCMs (1 mg/mL, 24 h) in the presence of NFκB inhibitor pyrrolidine dithiocarbamate (PDTC, 100 nm). (E) Quantification of calcium amplitude and (F) time to 50% baseline showed no difference between control and AGE + PDTC‐treated NRCM, indicating that NFκB inhibition suppressed the effect of AGE (*n* = 14 cells in each group).

It has previously been reported that RAGE activation by AGE may induce phosphorylation of members of the mitogen‐activated protein kinases (MAPKs) pathway, in particular the p38 kinase 21. Activation of this kinase modulates activation of the NFκB pathway 22. To investigate the involvement of these signalling molecules, we analysed the level of phosphorylated p38 kinase by western blots and activation of NFκB pathway following AGE treatment. Analysis of phosphorylated/total protein levels revealed that phosphorylation of p38 kinase was elevated in NRCM treated with AGE (Fig. 5A). Furthermore, assessment of NFκB activity using adenovirus‐delivered NFκB luciferase reporter construct showed a marked increase in NFκB activity following AGE treatment in a dose‐dependent manner (Fig. 5B). This finding was confirmed by immunofluorescence analysis, in which we found enhanced level of NFκB nuclear translocation in AGE‐treated NRCM, indicating activation of this transcription factor (Fig. 5C).

### The AGE‐induced Ca^2+^ transient change is NFκB dependent

We used a NFκB inhibitor pyrrolidine dithiocarbamate (PDTC) to test whether NFκB is required in mediating the AGE‐induced Ca^2+^ transient change in NRCM. NRCMs were treated with AGE (1 mg·mL^−1^) in the presence of 100 nm PDTC for 24 h. We observed that there was no difference in Ca^2+^ transient amplitude as well as Ca^2+^ decay rate between treated NRCM and control cells, indicating that PDTC suppressed the effect of AGE treatment in Ca^2+^ transient (Fig. 5D–F).

### iNOS expression and nitric oxide level are elevated following AGE treatment

**Figure 6:**
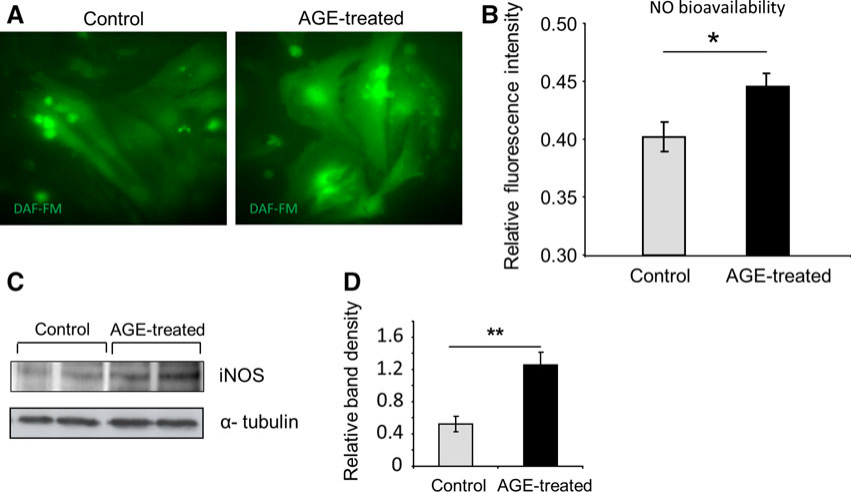
Nitric oxide (NO) bioavailability and iNOS expression are increased following AGE treatment. (A) Representative images of control and AGE‐treated NRCM stained with NO‐sensitive dye DAF‐FM. (B) Quantification of DAF‐FM fluorescent signal revealed a significantly higher NO bioavailability in AGE‐treated NRCM compared to control (*P* < 0.05, *n* = 94–106 cells). (C) Representative western blots and (D) quantification of band density showing expression of iNOS in control and AGE‐treated NRCM (*P* < 0.01, *n* = 7 in each group).

It has been reported that AGE accumulation is also related to nitric oxide (NO) production in kidney mesangial cells 23, endothelial cells 24 and macrophages 25. We therefore sought whether the level of NO bioavailability was altered in AGE‐treated cardiomyocytes. Using NO‐sensitive dye DAF‐FM, we observed significantly higher level of NO bioavailability in AGE‐treated cardiomyocytes compared to control (Fig. 6A,B).

Of three different isoforms of NO synthases, the inducible NO synthase (iNOS) is known to be modulated by p38 kinase and NFκB transcription factor 23. We therefore examined iNOS expression by western blot and found that iNOS expression was significantly elevated in AGE‐stimulated NRCM (Fig. 6C,D). These data suggested that iNOS expression might contribute to the elevation of NO bioavailability following AGE treatment.

### S‐Nitrosylation of calcium‐handling proteins in AGE‐treated NRCM

**Figure 7:**
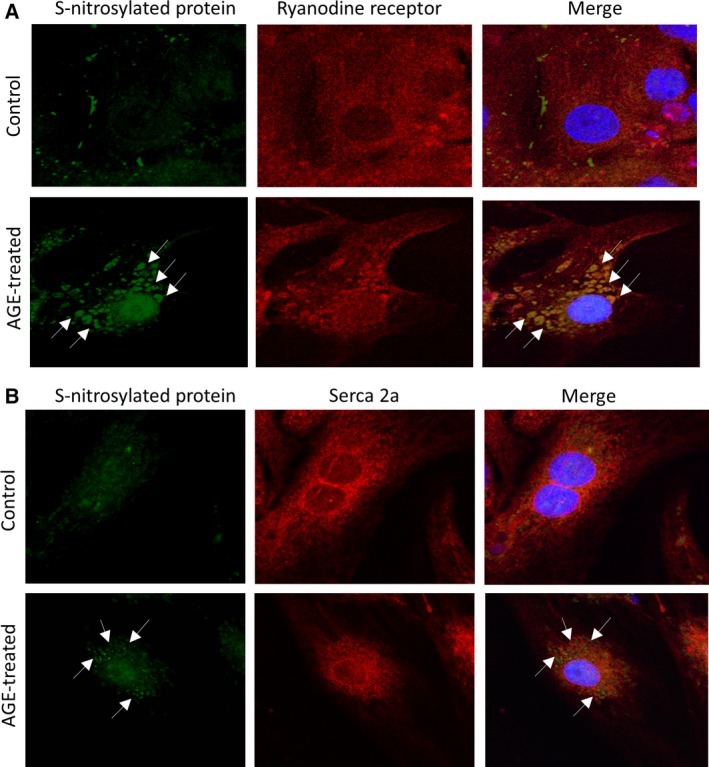
S‐Nitrosylation of ryanodine receptor and SERCA2a in AGE‐treated NRCM. (A) Representative immunofluorescence detection of S‐nitrosylated protein (green), ryanodine receptor (red) and the merged images showing increased S‐nitrosylation in AGE‐treated cells and the colocalization of S‐nitrosylation signal (arrows) with ryanodine receptor. (B) Similar analysis was also performed to detect S‐nitrosylation of SERCA2a. S‐Nitrosylation (green) was increased in AGE‐treated cells and colocalization with SERCA2a (red) was also detected although at a lower level than that observed with the ryanodine receptor. Nuclei were stained blue with DAPI.

Increased production of both NO and superoxide may induce the formation of peroxynitrite that can cause nitrosylation of thiol groups of particular proteins and hence impair their functions 26, 27. As an initial step to test whether the increased ROS/NO generation shown above induced S‐nitrosylation of major Ca^2+^ channels in AGE‐treated cardiomyocytes, we analysed S‐nitrosylation of ryanodine receptor and SERCA2a in AGE‐treated cells. For this purpose, we performed immunofluorescence analysis followed by detection using confocal microscopy. As shown in Fig. 7A,B, we found substantial colocalization between S‐nitrosylated protein and the ryanodine receptor, and to a lesser extent with the SERCA2a. Together, these data suggest that S‐nitrosylation of calcium‐handling proteins may be a contributing factor in causing the reduced SR content and amplitude of calcium transient after AGE treatment.

## Discussion

Advanced glycation end products (AGEs) have been known to play important roles in inducing diabetes complications in many organs including heart. In this study, we reveal signalling pathways that may be involved in mediating AGE effects in cardiomyocytes, in particular in the regulation of intracellular Ca^2+^ homeostasis. As intracellular Ca^2+^ is key in determining contractile function, our finding may explain the molecular events leading to the development of cardiomyopathy in patients with diabetes.

We used neonatal rat cardiomyocytes (NRCM) as a model in this study as these cells express the receptor of AGE (RAGE). We found that AGE treatment markedly reduced Ca^2+^ transient amplitude, Ca^2+^ decay rate and the SR Ca^2+^ content. Our data are consistent with previous observation showing that AGE treatment reduces SR Ca^2+^ content and Ca^2+^ amplitude in cardiomyocytes 28. However, in addition to that finding, we also showed in this study that the expression levels of major Ca^2+^ regulators were not altered, suggesting that AGE modified the function rather than the expression of Ca^2+^ channels.

We also found signalling events induced by AGE treatment in NRCM, which might be related to each other and might contribute to the AGE‐induced alteration of Ca^2+^ homeostasis: (a) elevation of reactive oxygen species (ROS), (b) induction of NADPH oxidase activity, (c) activation of p38 kinase and NFκB and (d) induction of iNOS expression and NO level.

Oxidative stress is a key factor in the pathophysiology of diabetic complications 29 **.** RAGE activation following binding with AGE has been shown to mediate oxidative stress in a number of tissues including vascular 30, 31, renal 32, 33 and neuronal tissues 34. Our data showing that AGE‐treated cardiomyocytes contain higher level of ROS are in line with previous observation 28, 35. It is also consistent with the idea that RAGE activation induces ROS production 36. However, in this study, we also demonstrated the possible source of ROS elevation. We found that NADPH oxidase activity was markedly increased in AGE‐treated cardiomyocytes. Indeed, activation of NADPH oxidase by AGE has been observed in different cell types such as hepatic cells 19 and renal mesangial cells 37. Importantly, our data also showed that the induced NADPH oxidase activity might be responsible for reducing the Ca^2+^ transient in AGE‐treated cells as we found that NADPH oxidase inhibition using DPI normalized Ca^2+^ transient amplitude to basal levels.

To further examine the downstream signalling pathway, we analysed activation of p38 kinase as this kinase is part of the major stress pathway (the MAPK pathway) and has been associated with the NADPH oxidase‐derived ROS 38. Our findings showed a significant increase in p38 phosphorylation that indicated activation of this kinase. Indeed, AGE has been associated with activation of MAP kinase pathway in cardiomyocytes previously 35. This is also consistent with published observations in different cell types, which showed activation of p38 kinase by NADPH oxidase and ROS 39, 40. However, in this study, we also show the signalling pathway downstream of p38 that might be activated by AGE treatment. We observe activation and nuclear translocation of the transcription factor NFκB. Using luciferase reporter system and then confirmed by immunofluorescent staining, we detected higher NFκB activity and more nuclear NFκB in the AGE‐treated cardiomyocytes. NFκB activation might be associated with p38 kinase as it has been known that p38 kinase controls NFκB activation and nuclear translocation in various cell types 22, 41, 42. Our present findings confirmed that this regulatory process also occurs in cardiomyocytes in the setting of AGE exposure. Equally important, we also confirmed that the NFκB signal contributed to the reduction in Ca^2+^ dynamic, as indicated by the inhibitor experiments, adding to the functional importance of the pathway in the context of high AGE exposure.

We then investigated the downstream effector of NFκB activation by AGE in cardiomyocytes. We focused on NO signalling, in particular those regulated by inducible NOS (iNOS). This is because (a) RAGE activation upregulates NO production by iNOS 43; (b) iNOS is known as a gene target of NFκB transcription factor 23; (c) NO is a major regulator of Ca^2+^ transient in cardiac myocytes 44, 45, 46. Our data suggested that in cardiac myocytes AGE treatment might induce iNOS upregulation, which in turn increased intracellular NO level. Although at physiological levels NO may be beneficial, it is believed that supraphysiological concentrations of NO may inhibit the activity of specific Ca^2+^ channel such as ryanodine receptor 47. Furthermore, simultaneous enhanced intracellular generation of both NO and superoxide above the physiological levels may cause excessive nitrosylation of nonspecific cysteine residues, a process known as poly‐S‐nitrosylation 26. S‐Nitrosylation has been shown to modulate the function of major calcium regulator proteins such as SERCA, ryanodine receptor and L‐type Ca^2+^ channel (reviewed in 27). Physiological level of S‐nitrosylation is needed for optimum function of the Ca^2+^ channel; for example, normal nitrosylation level will activate ryanodine receptor opening 48 but too much nitrosylation will inhibit its function 47. Our initial assessment of S‐nitrosylation levels of ryanodine receptor and SERCA2a supports this idea as we observed increased levels of S‐nitrosylated proteins which were colocalized with ryanodine receptor and SERCA2a in NRCM treated with AGE.

**Figure 8:**
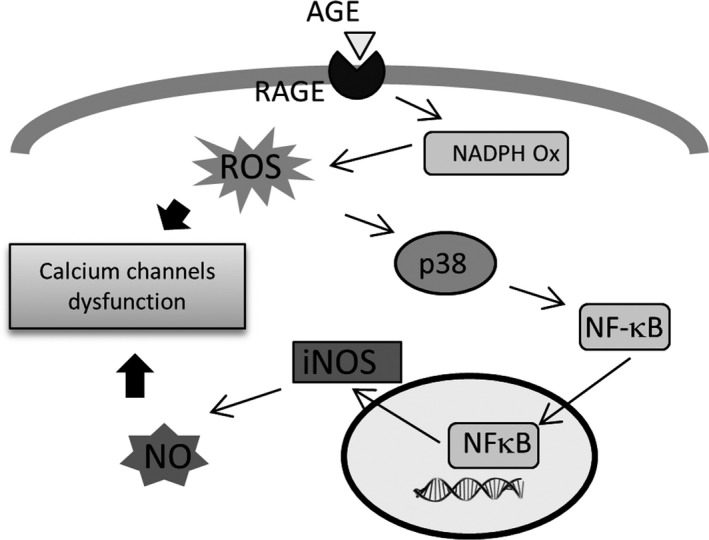
Schematic diagram showing the signalling pathway activated by AGE treatment in cardiomyocytes. RAGE activation by AGE leads to the elevation of ROS and NO via activation of NADPH oxidase and the p38 kinase/NFκB pathway. Elevated ROS and NO may interfere with the functions of calcium‐handling proteins, likely in part due to S‐nitrosylation.

Taken together, our data support the presence of a pathway in cardiomyocytes that is regulated by AGE treatment and may be important in modulating the intracellular Ca^2+^ transient. AGE‐induced activation of RAGE may stimulate NADPH oxidase and hence production of ROS. This may activate p38 kinase, in turn activating NFκB and promoting its nuclear translocation. This may then induce iNOS expression eventually resulting in increased intracellular NO levels, which together with the increased ROS may then alter Ca^2+^ handling through S‐nitrosylation of key proteins (Fig. 8).

In summary, our study has identified signalling molecules that may be involved in mediating the effects of exposure of advanced glycated end products (AGEs) in cardiomyocytes. The data will add to the growing body of evidence on the crucial roles of AGE in mediating diabetes complications. It also underlines that targeting component(s) of this signalling axis may be useful to prevent the development of AGE‐induced cardiomyocyte dysfunction.

## Author contributions

ZH performed experiments and analysed data. TM performed and helped designing experiments. NS performed experiments. MM designed and supervised the project. EJC supervised the project. DO helped designing experiments, analysed data and wrote the manuscript.
